# Posterior Reversible Encephalopathy Syndrome: A Case Report With Contemporary Literature Review and Neuropathological Evidence From Autopsy

**DOI:** 10.7759/cureus.87832

**Published:** 2025-07-13

**Authors:** Liubou Kazacheuskaya, Jordan Brzezny, Luis De Alba, Marjorie R Fowler, James G Traylor

**Affiliations:** 1 Pathology and Translational Pathobiology, Louisiana State University Health Sciences Center, Shreveport, USA; 2 Radiology, Louisiana State University Health Sciences Center, Shreveport, USA; 3 Radiology, Willis Knighton Health, Shreveport, USA

**Keywords:** antisynthetase syndrome, autopsy, axonal swelling, neuropathology, posterior reversible encephalopathy syndrome (pres), post hsv anti-nmda receptor encephalitis, vasogenic brain edema, white matter edema

## Abstract

Posterior reversible encephalopathy syndrome (PRES) is a neuro-radiological condition characterized by vasogenic edema in the parieto-occipital brain regions. Although typically reversible, some cases result in irreversible damage, particularly with delayed treatment. This study reviews 12 autopsy-confirmed cases and presents a novel case, emphasizing neuropathological findings that challenge the assumption of reversibility. Common pathological features include white matter edema, axonal swelling, and fibrinoid necrosis.

Risk factors for PRES include hypertension (HTN), autoimmune diseases, renal failure, immunosuppressive therapies, and organ transplantation. Clinical presentations range from headaches and seizures to coma, while MRI often shows T2 hyperintensities in posterior brain regions, which may resolve in follow-up imaging. Electroencephalogram (EEG) in the presented case demonstrated moderate diffuse slowing and some focal abnormalities, suggestive of cerebral dysfunction related to PRES.

This novel case involves a 27-year-old homeless African American woman with a history of autoimmune disease, who presented with pain, weight loss, and weakness. Initial findings included elevated inflammatory markers and antibodies, leading to diagnoses of polymyositis, kidney injury, and pericardial effusion. During hospitalization, she experienced seizures and was suspected of having anti-NMDA (anti-N-methyl-D-aspartate) receptor encephalitis, for which she received IV immunoglobulins. MRI findings aligned with PRES. Despite interventions, including plasma exchange and rituximab therapy, she experienced cardiac arrest and ultimately passed away.

Autopsy data provides crucial insights into the progression of PRES, emphasizing the importance of prompt diagnosis and treatment to prevent irreversible damage and fatal complications.

## Introduction

Posterior reversible encephalopathy syndrome (PRES) is a relatively newly described condition that was first reported in 1996 after a series of patients were observed to have characteristic CT and MRI findings, including subcortical edema without infarction [1].

Today, PRES is a well-established clinicoradiological diagnosis, although histopathological reports remain limited. Known risk factors include severe hypertension (HTN), preeclampsia and eclampsia, autoimmune conditions, drug or alcohol intoxication, renal failure, immunosuppressive therapy, and solid organ or bone marrow and stem cell transplantation. Clinical presentations vary greatly among patients, ranging from mild, nonspecific symptoms such as headache, nausea, vomiting, and blurred vision to seizures, cortical blindness, encephalopathy, focal neurological deficits, and even coma [2,3]. The differential diagnosis includes hemorrhagic stroke, encephalitis, intracranial venous thrombosis, intoxication, migraine, and meningitis [4].

Neuroimaging plays a crucial role in the diagnosis of PRES. Classically, it presents as bilateral vasogenic edema in the posterior parietal and occipital lobes [5,6]. Radiographically, this appears as bilateral, symmetric regions of T2 and FLAIR white matter hyperintensities with corresponding hypointensities on T1 sequences. Restricted diffusion is not typically seen on diffusion-weighted imaging (DWI) or apparent diffusion coefficient (ADC) maps [7]. Although predominantly involving the parieto-occipital lobes, a significant minority of PRES cases also demonstrate involvement of atypical locations. These may include the frontal lobe, temporal-occipital region, cerebellum, and brainstem [8]. Among pathological findings described in autopsy-recovered brain parenchyma, the following changes were noted: white matter edema without evidence of fibrinoid necrosis or microinfarction, consistent with MRI findings of vasogenic edema. In this case, EEG (electroencephalography) performed during hospitalization revealed moderate diffuse slowing and focal cortical dysfunction in the bilateral posterior regions, supporting MRI and clinical impression of PRES. However, susceptibility-weighted imaging (SWI) was not included in the MRI protocol in our case.

It is worth mentioning that the name of the syndrome is partially misleading, as there are reported cases with non-reversible lesions in the brain and persistent clinical deficits, which may be attributed to a lack of timely or adequate therapy. Delay in appropriate diagnosis or treatment may influence recovery or even result in death [4,9,10].

## Case presentation

A 27-year-old homeless African American female initially presented to a medical center in April 2024 with complaints of generalized pain, significant unintentional weight loss, and progressive weakness that had begun approximately one year earlier. Her past medical history was notable for anxiety, depression, syphilis (treated with penicillin), and posttraumatic stress disorder due to sexual abuse. During evaluation, laboratory tests revealed abnormal inflammatory and autoimmune markers (Table 1). She was eventually diagnosed with polymyositis and found to have a pericardial effusion. Pericardiocentesis was performed, and she was discharged to rehab on colchicine, metoprolol, and prednisolone. Her condition deteriorated, and she began experiencing dyspnea on exertion and palpitations, leading to the creation of a pericardial window. Due to concern for an underlying autoimmune process driving her condition, she was transferred to a university hospital for a rheumatology evaluation.

**Table 1:** Summary of key laboratory findings with corresponding reference ranges.

Test	Result	Reference Range
Antineutrophilic antibodies	>1.8	Negative
Rheumatoid factor (RF)	28 IU/mL	≤ 15 IU/mL
Erythrocyte sedimentation rate (ESR)	50 mm/hr	0–20 mm/hr
C-reactive protein (CRP)	6.64 mg/dL	≤ 0.9 mg/dL
Blood pH	7.286	7.35–7.45
CSF protein	76 mg/dL	15–40 mg/dL
Anti-PL-12 antibody	Positive	Negative
SSA 52 kD antibodies	>8 AI	<1 AI
Lactate dehydrogenase (LDH)	622 U/L	110–260 U/L
Reticulocyte count	4.60%	0.5–2.5%
Haptoglobin	8 mg/dL	30–250 mg/dL
ADAMTS13 activity	53%	≥ 70%

At the hospital, after initial assessment, the patient was diagnosed with acute kidney injury, proteinuria, and metabolic acidosis (Table 1). Her clinical course included treatment for sepsis with vancomycin, cefepime, and metronidazole, as well as evaluation for colitis/enteritis (infectious vs. inflammatory) and concern for heparin-induced thrombocytopenia, which was treated with argatroban and later discontinued due to gastrointestinal bleeding. Lumbar puncture revealed abnormal cerebrospinal fluid findings (Table 1). Autoantibody testing supported a diagnosis of antisynthetase syndrome, and she was treated with Solu-Medrol and hydroxychloroquine.

**Figure 1:**
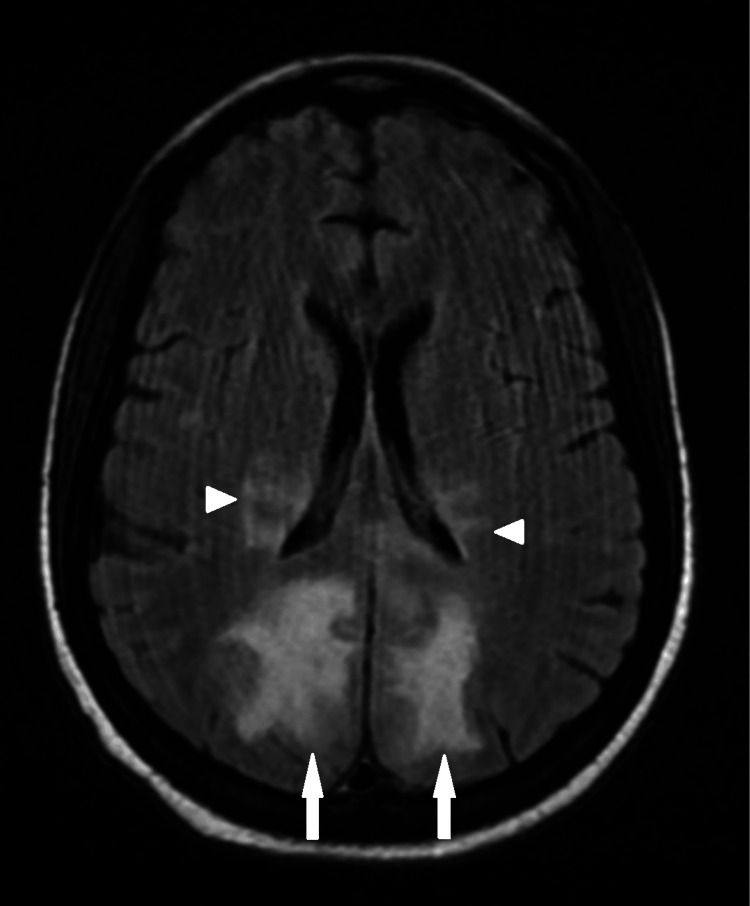
Axial FLAIR demonstrates white matter hyperintensities in the posterior parietal (arrowhead) and occipital lobes (arrow) that spare the gray matter.

**Figure 2:**
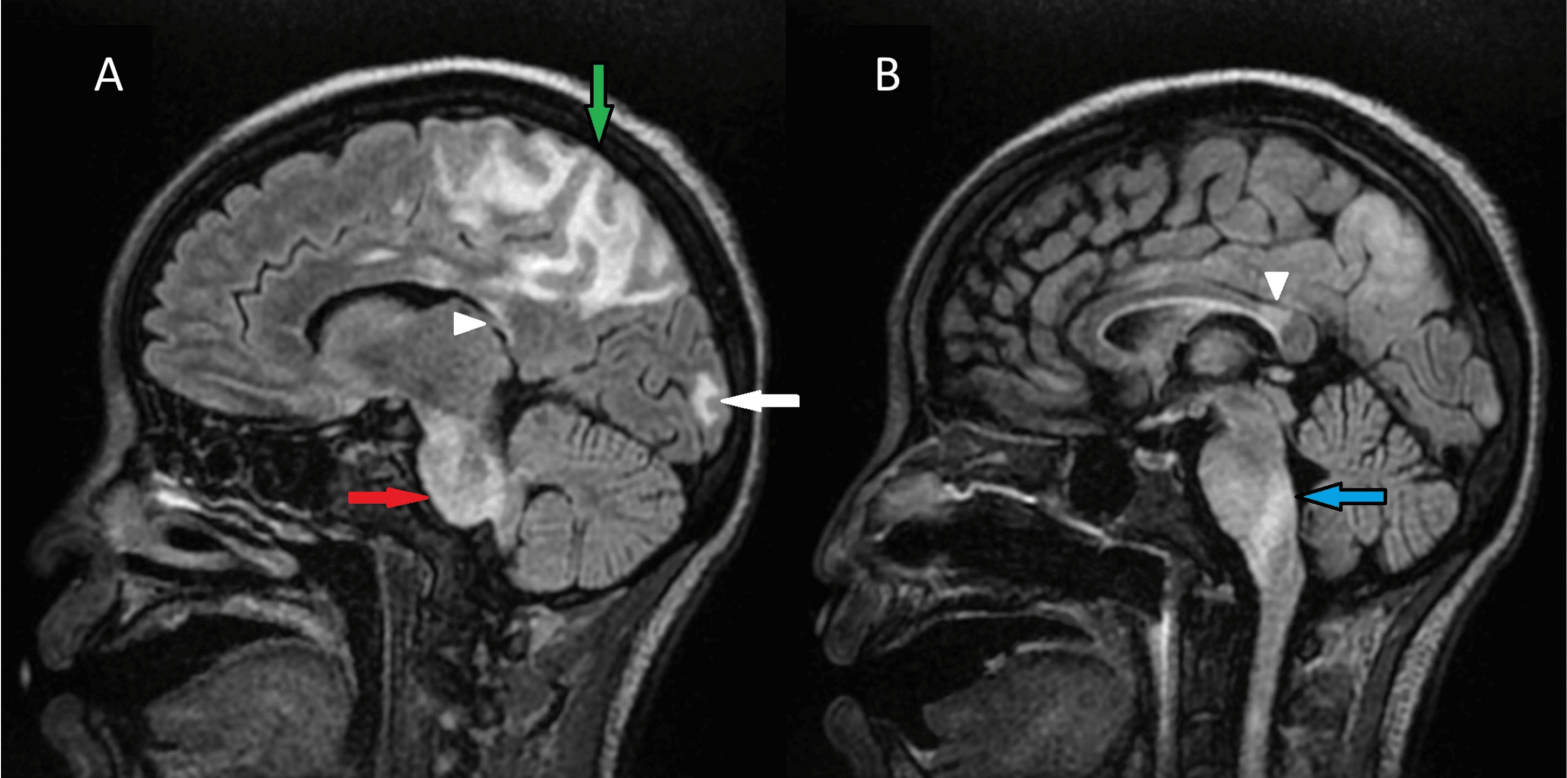
Left parasagittal FLAIR image demonstrates white matter hypointensities involving the parietal lobe (green arrow), occipital lobe (white arrow), corpus callosum (arrowhead), medulla (blue arrow), and pons (red arrow) (A). Sagittal FLAIR image better demonstrates involvement of the callosal isthmus (arrowhead) and medulla (blue arrow) (B).

During hospitalization, the patient experienced two episodes of seizures, leading to suspicion of anti-N-methyl-D-aspartate (NMDA) receptor encephalitis and treatment with intravenous immunoglobulins. EEG demonstrated moderate diffuse slowing, suggestive of moderate diffuse disturbance in cerebral function, and focal slowing in the bilateral posterior head regions, indicating focal cortical dysfunction. Brain MRI with and without contrast was obtained on a 1.5 Tesla magnet. Multiple white matter hyperintensities were noted, predominantly involving the posterior parietal and occipital lobes (Figure 1). However, involvement of other atypical regions was also noted (Figure 2); white matter hyperintensities were observed in the posterior frontal lobes, isthmus of the corpus callosum, pons, and medulla. No evidence of restricted diffusion or abnormal contrast enhancement was seen. Given this distribution and the preceding hypertensive state, these findings were compatible with posterior reversible encephalopathy syndrome with a variant central component.

Thrombotic thrombocytopenic purpura was suspected based on peripheral smear findings and laboratory abnormalities (Table 1). The patient completed four therapeutic plasma exchange procedures and was started on rituximab therapy. A mature cystic teratoma of the right ovary was incidentally noted on an abdominal/pelvic CT scan. The patient experienced multiple episodes of cardiac arrest and required significant hemodynamic support. Despite continued resuscitative efforts, the patient expired. Authorization for an unrestricted autopsy was granted.

**Figure 3:**
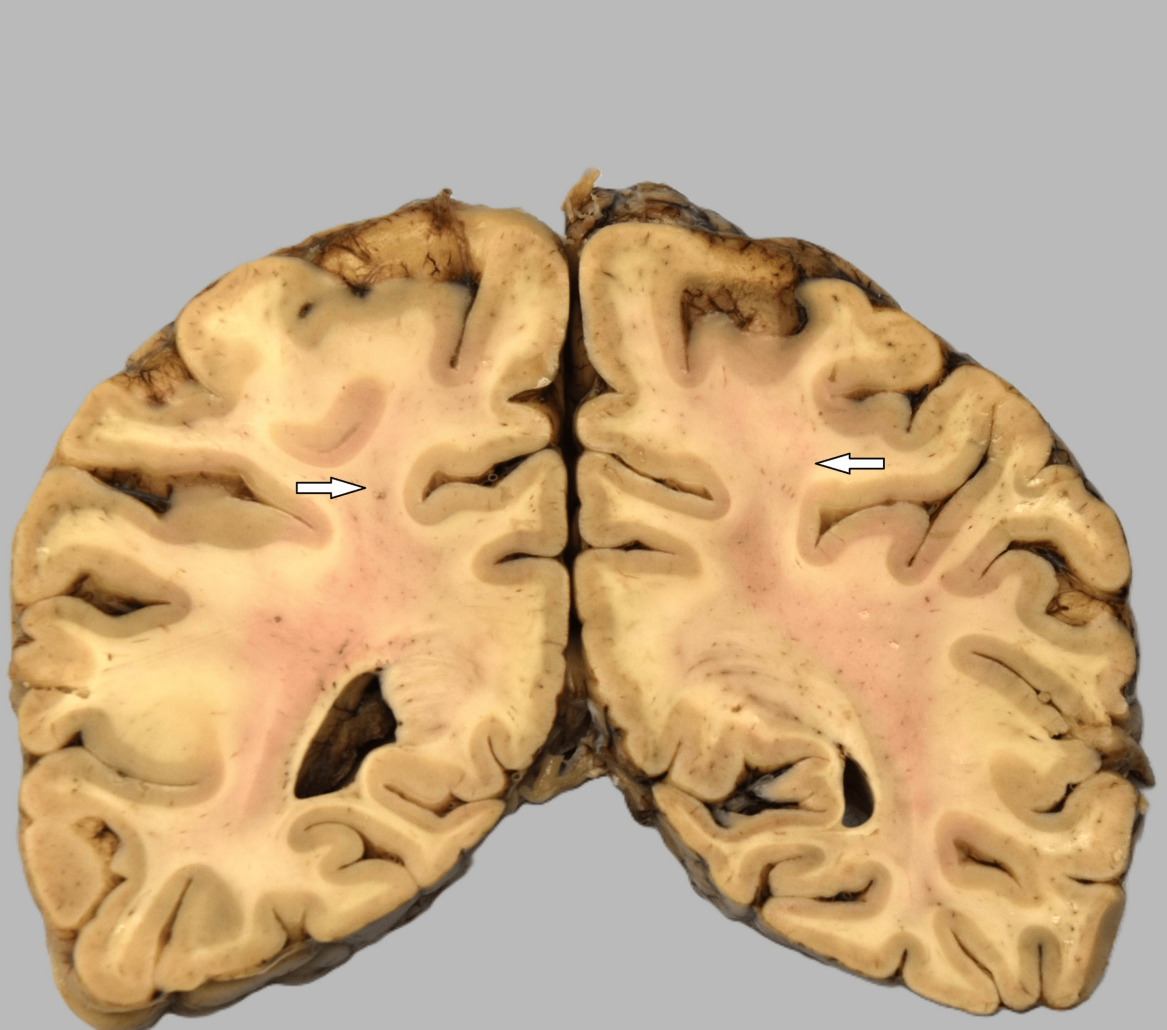
Gray discoloration of white matter (arrow) with spared subcortical U-fibers.

**Figure 4:**
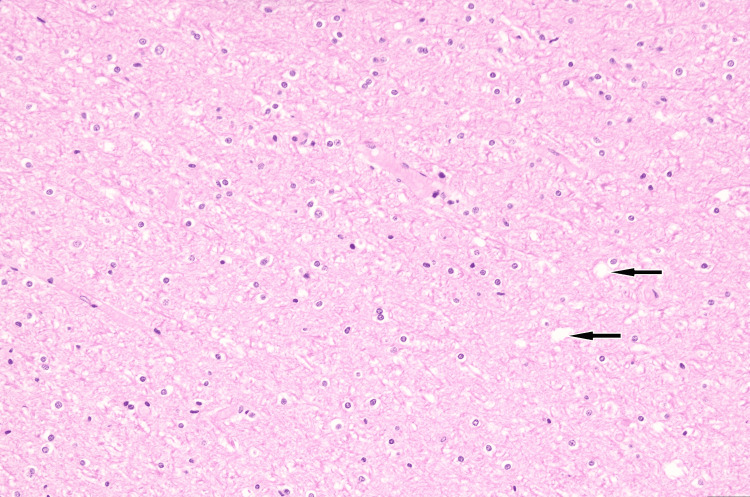
Sections stained with H&E of the right and left parieto-occipital areas showed patchy vacuolation of the neuropil (arrow) that spared the subcortical U-fibers 20X.

**Figure 5:**
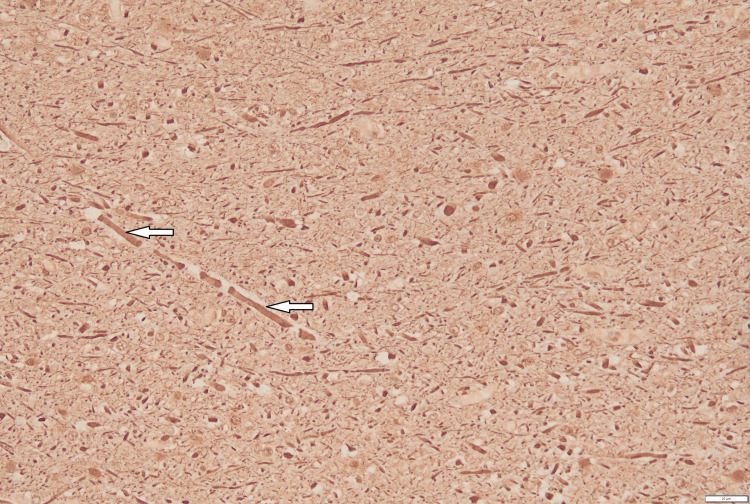
Neurofilament stain of enlargement of scattered axons (arrow). These areas also showed enlargement of scattered axons (6.8-8.5 microns) when compared to normal axons (3.4-5.1 microns) in the subcortical area. These swollen axons were frequently surrounded by clear vacuoles.

General autopsy findings from the kidney sections revealed occasional arteriolosclerosis, hemolyzed red blood cells within the glomeruli consistent with endothelial injury, and thickened vascular walls with an “onion-skin” appearance. The unfixed brain weight was recorded as 980 grams. The leptomeninges were thin and transparent. Sequential coronal sections of the parieto-occipital regions of the brain showed gray discoloration of the white matter, which spared the subcortical U-fibers (Figure 3). Sequential sagittal and parasagittal sections of the cerebellum demonstrated unremarkable folia and white matter. The brainstem was unremarkable. Sections through the right and left parieto-occipital areas showed patchy vacuolation of the neuropil that spared the subcortical U-fibers (Figure 4). These areas also showed enlargement of scattered axons (6.8-8.5 microns) compared to normal axons (3.4-5.1 microns) in the subcortical area. These swollen axons were frequently surrounded by clear vacuoles, best seen in sections stained with a neurofilament stain (Figure 5). Blood vessels stained with a CD31 immunostain appeared normal. Reactive astrocytes and macrophages were not seen. There were only rare lymphocytes.

## Discussion

Posterior reversible encephalopathy syndrome (PRES) was first described in 1996 as a condition with sudden neurological symptoms and characteristic brain imaging findings of subcortical vasogenic edema without infarction [1]. Since then, it has been reported in many different clinical settings. Known risk factors include uncontrolled high blood pressure, kidney failure, autoimmune diseases, eclampsia or preeclampsia, immunosuppressive drugs, chemotherapy, and organ or stem cell transplants [2-4].

Patients with PRES can have very mild to very severe symptoms. Common signs are headache, vision changes, seizures, confusion, weakness, and sometimes coma [2,3]. Brain imaging is very important to diagnose it. The usual pattern is symmetric T2 and fluid-attenuated inversion recovery (FLAIR) hyperintensities in the parieto-occipital white matter, with the cortex mostly spared and no restricted diffusion [5-7]. However, other brain areas can be involved, like the frontal lobes, brainstem, cerebellum, or deeper structures, which can make the diagnosis more difficult [8,9].

Although the name suggests that this condition is reversible, many reports now show that this is not always true. If diagnosis is delayed or the underlying cause is not treated quickly, patients can develop infarction, bleeding, necrosis, and permanent brain damage [10-12]. The autopsy cases reviewed in Tables 2, 3 show that the severity of damage can range from mild edema to severe tissue injury and necrosis.

**Table 2:** Compilation of clinical summary from revied case reports.

Gender	Age	Outcome	Clinical History	Medications	Source, Year (Corrected Reference #)
M	61	Death	B-cell Acute lymphoblastic leukemia, tumor lysis syndrome	Prednisolone, cyclophosphamide, vincristine, doxorubicin	Greenwood MJ et al. [11], 2003
M	60	Recovery	HTN, status post bilateral renal transplant	N/A	Schiff D et al. [12], 2005
F	70	Death	Multiple myeloma, thrombocytopenia	Melphalan, Vincristine, Cyclophosphamide, Adriamycin, Prednisolone	Okeda R et al. [13], 2007
F	26	Death	Myeloid sarcoma of breast, s/p BMT	Idarubicin, cytarabine, fludarabine, melphalan, antithymocyte globulin, tacrolimus, prednisolone, total body irradiation	Hayashi Y et al. [14], 2017
M	74	Recovery	HTN	N/A	Decker D et al. [15], 2009
F	8	Death	Pre-B-cell acute lymphoblastic leukemia, polyserositis	Chemotherapy and total body irradiation therapy (1350 cGy) followed by matched cord-blood stem cell transplant. Cyclosporine, prednisone, amlodipine	Kheir JN et al. [16], 2010
F	62	Clinical recovery from PRES, death 4.5 years later	Systemic lupus erythematosus (SLE), HTN	Cyclophosphamide and hydroxychloroquine	Jacquot C et al. [17], 2015
F	24	Recovery from PRES, death 3 months later	Stem cell transplantation for acute myeloid leukemia	N/A	Singer S et al. [18], 2015
M	48	Recovery from PRES, death 4 months later	Stem cell transplantation for refractory chronic myeloid leukemia	N/A	Singer S et al. [18], 2015
F	57	Death	HTN	Methylprednisolone	Ismail FS et al. [10], 2021
F	88	Death	Myelodysplastic syndrome, HTN	Darbepoetin alfa	Takigawa K et al. [19], 2024
M	48	Death	Status post liver transplantation, HTN, TTP	Cyclosporin	Easton A [20], 2006
F	27	Death	SLE, antisynthetase syndrome, sepsis	Colchicine, Lopressor, Prednisolone	Current study

**Table 3:** Compilation of neuropathologic and image findings from reviewed case reports.

Images (MRI/CT)/Time After Symptom Onset	Repeat Images/Time	Time After Symptoms Onset/Location /Neuropathological Findings	Source (Corrected Reference #)
Cerebral CT (day 8): extensive bilateral low density white matter changes predominantly occipital and posterior parietal lobes. Mild mass effect with ventricular effacement posteriorly. No infratentorial involvement.	Cerebral CT (day 15): partial resolution of posterior low density white matter changes with reduced mass effect	Day 39/Posterior white matter: Multiple necrotic foci, gliosis, neuropil rarefaction, red cell extravasation, spheroids, lipid-laden macrophages consistent with ischemia.	Greenwood MJ et al. [11]
MRI: Multifocal subcortical T2 hyperintensities in bilateral parieto-occipital regions	Follow-up MRI: complete lesion resolution	5 days after symptom onset/Right parietal lobe: White matter with mild diffuse vacuolation and mild inflammatory reaction (scattered macrophages and rare lymphocytes). Abundant reactive astrocytes	Schiff D et al. [12]
MRI (Day 17): Multifocal high-signal areas cerebral white matter, most prominent right frontal lobe. CT (Day 23): Mild subdural hematoma. T2-weighted MRI (Day 27): Multifocal high-signal cerebral white matter	N/A	Day 39/All cerebral lobes: Vacuoles in deep white matter, astrocytic swelling, segmental axonal swelling	Okeda R et al. [13]
MRI: Diffuse T2 hyperintensities pons and midbrain, extending to internal capsules, bright DWI signal with increased ADC values	N/A	~2 weeks after onset/Right middle cerebellar peduncle: Fibrinoid necrosis in arterioles, lymphocytic perivascular infiltrate, axonal swelling	Decker DA et al. [14]
CT (headache onset): extensive low attenuation cerebellum with edema causing obstructive hydrocephalus. MRI (next day): extensive patchy T2 signal in cortex and subcortical white matter parietal, occipital lobes, cerebellum, less frontal lobes; increased diffusion; petechial hemorrhages	Repeat head CT: worsening cerebellar tissue displacement and petechial hemorrhages	1. Cerebellar tonsillar biopsies: macrophages in leptomeninges, reactive endothelial cells. 2. Autopsy occipital & posterior frontal white matter: endothelial prominence, vascular wall thickening, foamy macrophages, myelin pallor, dilated perivascular spaces with fibrin, vasogenic edema, focal fibrinoid necrosis and petechial hemorrhages in cerebellum	Kheir JN et al. [15]
CT: Confluent white matter low density mainly posterior parietal and occipital lobes. MRI: PRES consistent with extensive symmetric subcortical white matter T2 hyperintensity	Follow-up MRI (2.5 years later): patchy nonspecific white matter changes, resolution of previous lesions	4.5 years after onset/Frontal, parietal, occipital lobes: diffuse mild white matter rarefaction, rare, scattered lymphocytes	Jacquot C et al. [16]
N/A	N/A	3 months after PRES recovery/Cerebral white matter: mild diffuse astrogliosis	Singer S et al. [17]
N/A	N/A	4 months after PRES radiographic (CT only) recovery/Cerebral white matter: lamellated perivascular mineralization and macrophages around scattered blood vessels	Singer S et al. [17]
FLAIR MRI (Day 3): multiple bilateral hyperintensities posterior white matter and left corona radiata. DWI and ADC maps show lower and higher intensities respectively. T2-gradient echo: multiple cerebral hemorrhages	N/A	24 hours after MRI/Occipital white matter: cerebral edema, pale myelin sheaths, endothelial enlargement, plasma leakage, clasmatodendrosis	Hayashi Y et al. [18]
MRI: Bilateral symmetric extensive diffuse restriction with T2 hyperintense signals (frontal, parietooccipital, cerebellar, temporal) - full-blown PRES	N/A	Died during initial hospitalization/Cerebral and cerebellar hemispheres: extensive leukoencephalopathic changes, diffuse myelin pallor, mild astroglial activation, thin-walled vessels with fibrinoid necrosis, petechial hemorrhages	Ismail FS et al. [10]
CT (day 16): hypoabsorption bilateral cerebellar hemispheres. MRI (day 17): low T1 and high T2 signals, high diffusion, no ADC signal reduction	N/A	Day 23/Cerebrum and cerebellum: widespread cerebral white matter edema, cerebral hemorrhage, subarachnoid hemorrhage	Takigawa K et al. [19]
Admission CT: deep left intracerebral hematoma with surrounding edema. Occipital horns compressed by bilateral hypointense white matter lesions. Next day: uncal herniation, hemorrhage expansion, bilateral white matter unchanged	N/A	Initial hospitalization/Occipital white matter: acute left frontal hematoma, early infarction, zones of diffuse pallor sparing subcortical zones	Easton A [20]
CT (after first seizure): nonspecific cortical-subcortical hypodensity bilateral frontoparietal region. MRI (next day): confluent T2 FLAIR hyperintense white matter signal posterior frontal lobes, parieto-occipital lobes, pons, medulla, callosal isthmus	Follow-up MRI (day 7): findings consistent with PRES, significant interval improvement	Day 18 after first seizure/Right and left parieto-occipital areas: patchy neuropil vacuolation sparing subcortical U-fibers, enlargement of scattered axons	Current study

Looking at published cases, common findings at autopsy include white matter edema, vacuoles in the tissue, swollen axons, and, in worse cases, necrosis, damaged blood vessel walls (fibrinoid necrosis), and bleeding [11-20]. For example, Greenwood et al. [11] described multiple necrotic spots and gliosis in a leukemia patient with tumor lysis syndrome. Kheir et al. [15] reported a child with leukemia and a stem cell transplant who had severe vascular injury, fibrinoid necrosis, and small hemorrhages. These findings match the different outcomes seen in patients: some fully recover, but others die from worsening brain injury (Table 2).

In our patients, the disease had a severe course due to multiple risk factors happening at the same time: antisynthetase syndrome, sepsis, high blood pressure, and possible thrombotic microangiopathy. Her brain imaging showed the typical posterior white matter changes but also involved the brainstem, medulla, and corpus callosum, which fits with central-variant PRES [8]. At autopsy, there was clear vacuolation and axonal swelling in the white matter, but no big areas of necrosis or significant inflammation, which shows mostly vasogenic edema without major infarction.

EEG showed moderate diffuse slowing, indicative of a generalized cerebral dysfunction of uncertain etiology, along with focal slowing in the bilateral posterior head regions, suggestive of localized cortical involvement that might be compatible with PRES. Although susceptibility-weighted imaging (SWI) was not performed and microhemorrhages were not observed on autopsy, the addition of advanced MRI sequences such as SWI could enhance the detection of subtle hemorrhagic changes in future cases.

Compared to other reported autopsy cases, this patient’s brain did not have widespread necrosis, but it shows that even severe edema alone can be fatal when the body is already in multi-organ failure. This case shows once again that the term “reversible” can be misleading if the cause is not treated quickly. If the underlying disease keeps damaging the blood vessels and brain, PRES can become irreversible and lead to death [10,14,15].

Overall, this case supports that PRES can involve more than just the parieto-occipital regions and that the actual brain damage can vary from mild edema to severe necrosis and bleeding. Non-invasive diagnostic modalities, including EEG and advanced MRI sequences, might be helpful for early detection and guiding treatment to prevent irreversible brain injury. Early detection and prompt treatment of the underlying cause are key to preventing permanent injury. More studies with larger autopsy series are needed to better understand which patients are at higher risk for irreversible brain damage and to help guide timely treatment in complicated cases.

## Conclusions

Posterior reversible encephalopathy syndrome (PRES) is a complex disorder with diverse clinical and radiological manifestations. Although many cases resolve with timely treatment, others may progress to irreversible brain injury, particularly when multiple risk factors or delays in care are present. Autopsy findings from this and other cases (Tables 2, 3) reveal a spectrum of pathology, ranging from mild edema to severe necrosis and hemorrhage.

This case underscores that PRES can extend beyond the classic parieto-occipital regions and that reversibility is closely tied to early recognition and management. Without prompt intervention, outcomes can be poor, even with intensive supportive care. Furthermore, non-invasive diagnostic tools such as EEG and advanced MRI sequences (including susceptibility-weighted imaging) play an important role in early diagnosis and management, guiding timely treatment to prevent irreversible damage. Larger autopsy-based studies are needed to identify predictors of irreversible damage and to guide treatment strategies. Early diagnosis and aggressive therapy remain critical to preventing permanent injury or death from PRES.
